# Immunization with *Streptococcus suis* bacterin plus recombinant Sao protein in sows conveys passive immunity to their piglets

**DOI:** 10.1186/s12917-016-0937-8

**Published:** 2017-01-07

**Authors:** Kai-Jen Hsueh, Li-Ting Cheng, Jai-Wei Lee, Yao-Chi Chung, Wen-Bin Chung, Chun-Yen Chu

**Affiliations:** 1 Department of Veterinary medicine, College of Veterinary Medicine, National Pingtung University of Science and Technology, Pingtung, 91201 Taiwan; 2 Graduate Institute of Animal Vaccine Technology, College of Veterinary Medicine, National Pingtung University of Science and Technology, 1, Shuehfu Road, Neipu, Pingtung, 91201 Taiwan; 3 Department of Tropical Agriculture and International Cooperation, National Pingtung University of Science and Technology, Pingtung, 91201 Taiwan

**Keywords:** *Streptococcus suis*, rSao, Vaccine, Passive immunity, Cytokine

## Abstract

**Background:**

*Streptococcus suis* (*S. suis*) causes arthritis, meningitis, septicemia, and sudden death in pigs and is also an zoonotic agent for humans. The present study demonstrated that immunization with recombinant Sao-L (surface antigen one-L, rSao-L) protein from a strain of *S. suis* serotype 2 in pigs was able to increase cross-serotype protection against *S. suis* serotype 1 and 2 challenge. Since weaning piglets are more susceptible to *S. suis* infections due to the stresses associated with weaning, prepartum immunization in sows may convey passive immunity to piglets and provide protection.

**Results:**

Pregnant sows were immunized with a vaccine containing inactivated *S. suis* serotype 2 plus rSao as the antigens. Blood samples were collected from their piglets after birth for analysis of antigen-specific antibody titers and levels of various cytokines. Results demonstrated that the titers of *S. suis* and rSao-specific antibodies were significantly (*p* < 0.05) higher in the vaccinated piglets in comparison with that of piglets in the control group. The serum levels of interferon (IFN)-γ, interleukin (IL)-4, IL-6, and IL-12 were significantly (*p* < 0.05) increased in piglets born from vaccinated sows when compared to piglets from unvaccinated sows. In addition, piglets were challenged by heterologous and homologous *S. suis*. All piglets from unvaccinated sows developed severe symptoms of bacteremia, fever, anorexia, depression, and arthritis. On the other hand, piglets from vaccinated sows had significantly (*p* < 0.05) reduced clinical symptoms and lesion score (by 75 and 81%).

**Conclusions:**

Our results revealed that immunizing pregnant sows with the vaccine containing inactivated *S. suis* bacterin plus rSao as the antigens is able to enhance passive immunity against heterologous and homologous *S. suis* challenge in their piglets.

## Background

*Streptococcus suis* (*S. suis*) causes a spectrum of diseases, including arthritis, meningitis, septicaemia and sudden death, in pigs and is recognized as an important zoonotic agent for humans [1]. A total of 33 serotypes of *S. suis*, according to the structure of its capsular polysaccharides (CPS), have been identified [2]. Serotypes 1, 1/2, 2, 7, 9, 14 and 22 are more virulent than others and can be isolated worldwide, including the United States, Canada, Europe, and New Zealand [3]. In Taiwan, more than 80% of the clinical cases are *S. suis* positive [4], and most of them are infected with serotypes 1 and 2 [5]. The efficacy of many commercially available inactivated *S. suis* whole-cell vaccines left something to be desired due to the fact that the protection is only limited to homologous strains [6]. The CPS has been shown to play a pivotal role in the pathogenesis of *S. suis*, and that antibody against CPS (serotype 2) is essential for full protection from homologous challenge [7]. CPS is a poor immunogen due to its thymus-independent property and cannot induce sufficient immune response [8]. Study showed that coupling *S. suis* type 2 CPS to tetanus toxoid can induce T cell-dependent response, inducing IgM and IgGs for protection against challenges in mice and pigs [9, 10]. In addition, immunization with certain virulence factors, such as suilysin, muramidase-released protein (MRP) and extracellular factor (EF), has been demonstrated to protect pigs from challenge with homologous or heterologous strains of *S. suis*. However, discrepant results were reported when different virulence factors are expressed by the prevalent *S. suis* strains [11]. Therefore, researching for a more immunogenic antigen that is commonly expressed by most *S. suis* strains is imperative.

Many surface proteins are involved in the pathogenesis of Gram-positive bacteria and have been shown to elicit strong immune responses [12–14]. Immunization with recombinant *S. suis* SsnA protein (surface-anchored DNA-nuclease) formulated with aluminum hydroxide was able to protect mice from *S. suis* infection [15]. A new surface protein of *S. suis*, Abpb (amylase-binding protein B) is recently identified and immunization with recombinant Abpb provided effective protection against *S. suis* [16]. In addition, a Lam protein (CDS0330) was expressed on the cell surface of *S. sui*s 2 and was found to bind laminin *in vitro*. Immunization with recombinant CDS 0330 protein resulted in 50% survival rate of mice from *S. suis* 2 infection [17]. *S. suis*, Sao (surface antigen one), a common surface protein containing an Leu-Pro-X-Thr-Gly (LPXTG) motif, mediates many virulence factors during the course of infection. Sao is highly conserved among *S. suis* strains and has become a potential antigen for the development of effective vaccines against *S. suis* [13]. Sao protein is encoded by three allelic variants of gene with different lengths, Sao-S (1.5 kb), Sao-M (1.7 kb) and Sao-L (2.0 kb), respectively, and Sao-M is the most prevalent variant [14]. Immunization with rSao protein was able to elicit strong humoral antibody responses, decrease clinical signs and bacterial dissemination, increase survival rates, and confer cross-serotype protection in mouse and pig vaccination protocols [18], indicating that rSao is a suitable antigen for subunit vaccine development.

We previously demonstrated that immunization with recombinant Sao-L protein (rSao-L) from a strain of *S. suis* serotype 2 in pigs was able to increase antigen-specific antibody titers, the percentages of CD8^+^ and CD4^+^/CD8^+^ double-positive T cells, and cross-protection against *S. suis* serotype 1 heterologous challenge [19]. Since weaning piglets are more susceptible to *S. suis* infections due to the stress associated with weaning, prepartum immunization in sows may convey passive immunity to piglets and provide protection. This approach has been proven effective in preventing common swine diseases such as atrophic rhinitis [20], food-and-mouth disease [21] and classical swine fever [22]. Moreover, immunizing pregnant sows with a vaccine containing recombinant *Pasteurella multocida* toxin (rsPMT) plus *Pasteurella multocida* type A bacterin as the antigens significantly increased neutralizing antibody titer in colostrum when compared to pregnant sows immunized with the vaccine containing rsPMT only [20]. The combination of recombinant antigens with inactivated bacteria may provide extra antigens and elicit more broaden ranged protection in immunized animals. In the present study, pregnant sows were immunized with the vaccine containing inactivated *S. suis* serotype 2 plus rSao-L as the antigens. Passive immunity in their piglets was analyzed by examining serum antigen-specific antibody titers, levels of various cytokines, including interferon (IFN)-γ, interleukin (IL)-4, IL-6, IL-8, IL-12, and tumor necrosis factor (TNF)-α, and clinical signs after heterologous and homologous challenges with *S. suis* serotype 1 and 2.

## Methods

### Bacterial strains and expression of rSao

*S. suis* serotype 1 and 2 strains were obtained from the Pingtung County Animal Disease Control Center, Pingtung, Taiwan. Bacteria were grown in brain-heart infusion (BHI) broth supplemented with 0.5% yeast extract (Difco Laboratories, Spark, MD, USA) at 37 °C. The rSao was expressed as previously described [19]. The size of PCR product from the *S. suis* strain BCRC 14750 (ATCC 43765) was 2013 bp. Primers for rSao gene were designed according to GenBank accession no. JF 810176 (Sao-F: GCGGGAT CCATGAATACTAAGAAATGGAG and Sao-R: CAGAA GCTTGAACTAATTTACGTTTACGTG). The primers contained restriction enzyme (Bam HI/Hind III) cutting sites and the PCR product was cloned into the expression vector pET32a according to the manufacturer’s instructions (Novagen, Darmstadt, Germany). *E. coli* strain BL21 (DE3) harboring the recombinant plasmid was cultured in Luria-Bertanior modified medium (tryptone: 6 g/L, soytone: 1.5 g/L, yeast extract: 7.5 g/L, KH_2_PO_4:_ 1.2 g/L, K_2_HPO_4_: 0.6 g/L, glucose: 2 g/L) at 37 °C until the absorbance reached 0.6 at 600 nm. Isopropylthio-β-D-thiogalactose (IPTG) (Amresco, Ohio, USA) was added at a final concentration of 1 mM, and the culture was grown for 4 h with agitation. The resulting culture was ultrafiltrated by Vivaspin 20 100KDa MWCO (GE Healthcare, UK). The concentrated culture was analyzed by SDS-PAGE and Western blotting using the antisera from rabbits immunized with purified rSao. Contamination of endotoxin in the crude rSao was examined using Limulus Amebocyte Lysate test, LAL (CAPE COD, MA, USA) and the result was negative (no clotting, indicating that the concentration of endotoxin is less than 0.125 IU/mL).

### Purification of rSao

*E. coli* expressing rSao were centrifuged at 4 °C, 14,000 xg for 20 min. The pellet was resuspended in denaturing lysis buffer, sonicated using Misonix Sonicator S-4000 (Misonix, New York, USA), and filtered (0.22 μm). The rSao was purified using the ProfiniaTM Protein Purification System (BIO-RAD, California, USA) according to the manufacturer’s instructions. Urea was incrementally dialyzed out of the samples, from 6 M, 5 M, 4 M, 3 M, 2 M, and 1 M urea to 0.85% saline, using 12,000–14,000 MWCO Dialysis Membrane (Cellu•Sep® MFPI,Orange Scientific, Braine-l'Alleud, Belgium). Thereafter, BSA was used as the standard for determining the concentration of rSao.

### Preparation of vaccines

Formalin-inactivated *S. suis* serotype 2 bacteria and rSao in the aqueous phase final concentration in the vaccine for *S. suis* and rSao was 1x10^9^ CFU/mL and 100 μg/mL, respectively. The oil vaccine prepared with internal aqueous phase was emulsified with 25% of water-in-oil-in water (w/o/w) adjuvant containing two inactivated antigens of *S. suis* and rSao: oil phase: external aqueous phase ratios of 1:1:2 [19, 23]. The vaccine did not contain any viable microorganisms, which was confirmed by sterility test.

### Immunization of pregnant sows

Ten healthy pregnant sows, without previous exposure to *S. suis* (*S. suis* 2 antibody negative), were divided into two groups (*n* = 5 each). The vaccinated group was intramuscularly immunized with *S. suis* serotype 2 plus rSao vaccine (2 mL) 6 and 2 (boost) weeks before parturition. The other group was injected with an equal volume of sterile saline as the unvaccinated control. Animal experiments were conducted in a private pig farm in Tainan County, Taiwan, with informed consent from the owner to conduct research. The pigs were selected and randomly assigned to the treatment groups for the vaccine trial. All experimental protocols for animal trials were approved by the Animal Care and Use Committee, National Pingtung University of Science and Technology (NPUST). The experiments were conducted based on the Ethical Rules and Law of NPUST.

### Assessment of passive immunity in piglets

Blood samples (3–4 mL) were collected from the superior vena cava 2, 4, and 6 weeks after parturition from 5 healthy piglets randomly selected from each groups (*n* = 5) for determining the serum antibody titer and cytokine levels. Five weeks after parturition, 3 piglets were randomly selected from each group (*n* = 3) and challenged i.p. with 2 mL (1.06 × 10^9^ CFU/mL) of live *S. suis* serotype 1 (P1) or *S. suis* serotype 2 (P2) bacteria. The piglets were monitored daily for clinical signs, body temperature (fever was defined as anal temperature≧40 °C), and bacteremia. Seven days after the challenge, the piglets were i.m. injected with Stresnil (0.1 mL/kg), sacrificed, and dissected. Thereafter, pathological examination was performed and lesion score was calculated based on the area of lesions in an organ, where no lesion = 0, lesion area < 33% = 1, lesion area 33–66% = 3, lesion area > 66% = 5. The necropsy samples of various organs were cultured for bacteriological analysis.

### Indirect enzyme-linked immunosorbent assay (ELISA) for serum antibody titers

Flat-bottomed 96-well plates were coated with either *S. suis* serotype 2 bacteria (1.25 μg/mL) or purified rSao (1.25 μg/mL) in the coating buffer (15 mM Na_2_CO_3_, 35 mM NaHCO_3_, 3 mM NaN_3_, pH = 9.6) at 4 °C overnight. The serum antibody titers were determined as described [19], where S/P ratio = (Sample – NCˉ x) / (PCˉ x – NCˉ x) (NC: negative control, PC: positive control). To obtain positive serum, *S. suis*-negative SPF pigs were challenged with 2 ml (1 × 10^9^ CFU/ml) *S. suis* 2 and serum was collected 14 days later.

### Detection of serum cytokine levels

The serum cytokine levels, including IFN-γ, TNF-α, IL-4, IL-6, IL-8 and IL-12, were analyzed using the MILLPLEX® MAP Kit (EMD Millipore Corporation, Billerica, MA, USA) according to the manufacturer’s instructions. The plates were read by MAGPIX® plate reader at 470–565 nm (EMD Millipore Corporation).

### Detection of bacteremia by multiplex PCR

The detection of bacteremia in blood and organ samples after challenge was carried out using bacteriology and the Multiplex PCR based method. Primers for *S. suis* cps II and Sao gene were designed by Smith [24] and Hsueh [19], respectively: (cps II-F: GGCGGTCTAGCAGATGCTCG and cps II-R: GCGAACTGTTAGCAATGAC) (Sao-F: GCGGGATCCATGAATACTAAGAAATGGAG and Sao-R: CAGAAGCTTGAACTAATTTACGTTTACGTG). Bacterial DNA was isolated using the Blood & Tissue Genomic DNA extraction Miniprep System kit (Viogene, Sunnyvale, CA, USA). The genes were amplified by 30 PCR cycles consisting of 1 min denature at 94 °C, 2 min annealing at 56 °C and 2 min elongation at 72 °C.

### Statistical analysis

All data were expressed as mean ± S.E.M. and were compared using Student’s *t*-test. A *p*-value of < 0.05 was considered significant.

## Results

### Incidence of fever and bacteremia after challenge

**Fig. 1:**
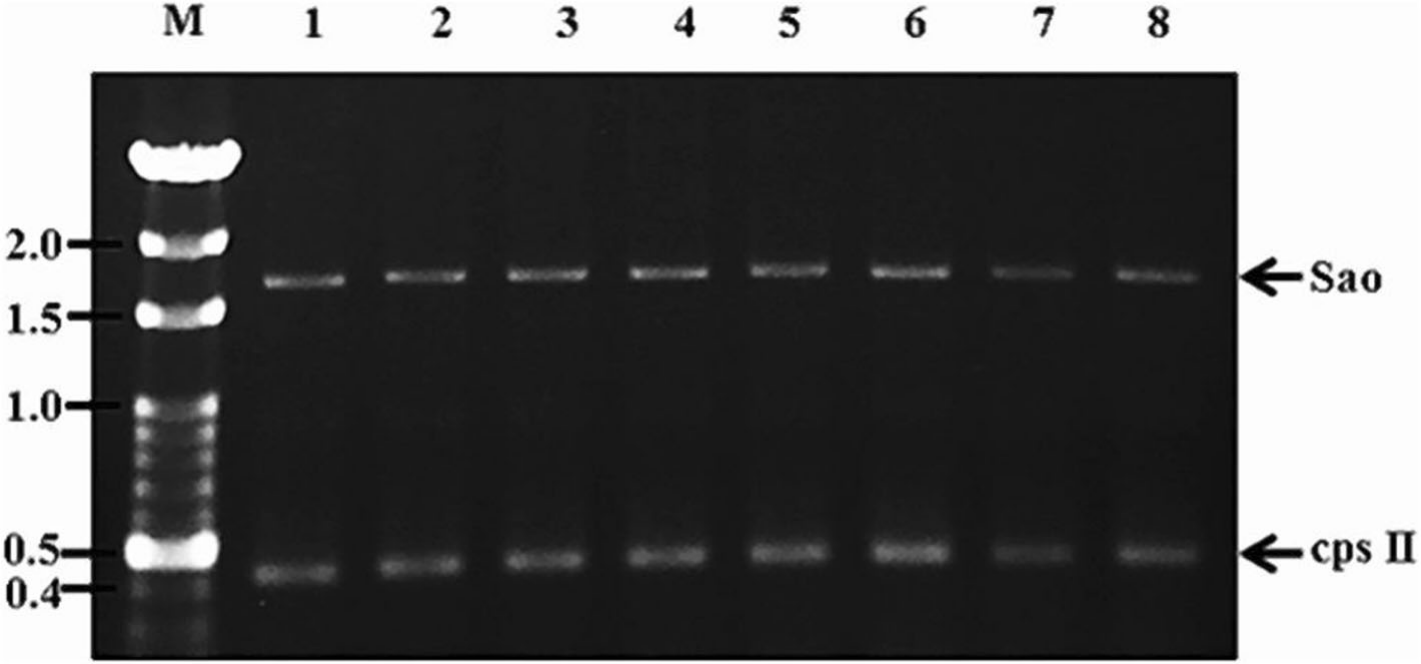
Detection of Sao and cps II genes in bacteremia samples by multiplex PCR. M: 100 bp DNA Ladder; Lane 1–6: n of 3 on day 1 and 2 after challenge as control group; Lane 7: Vaccinated group, two days after challenge; Lane 8: *S. suis* serotype 1 as the positive control

Two piglets from the control group had fever on the first (≧40 °C) and second (≧41 °C) day after challenge. One of the piglets with fever was confirmed to have bacteremia and died on the third day after challenge. In contrast, all piglets from the vaccinated group remained healthy and no fever was observed (Table 1). Results of multiplex PCR demonstrated that all the piglets in the control group had bacteremia during the first two days after challenge (Fig. 1). On the other hand, only one piglet in the vaccination group had bacteremia on the second day (Table 1) and seventh day (Table 2) after challenge. The difference between the vaccinated and the control group was marginal significant (*P = 0.058* and *P* = 0.140) (Tables 1 and 2).

**Table 1:** Fever and bacteremia of piglets after challenge with heterologous *Streptococcus suis* serotype 1 bacteria

Group^a^	Cases of fever/ bacteremia^b^ (%)							
	Day 0	1	2	3	4	5	6	7
Vaccination^c^	0/0	0/0	0/33	0/0	0/0	0/0	0/0	0/0
Control	0/0	33 ^d^/100	33 ^e^/100	0/0 ^f^	0/0	0/0	0/0	0/0

^a^Vaccination: *S. suis* 2 + rSao, Control: sterile saline

^b^The fever and bacteremia of piglets were monitored daily for 7 days after challenge

^c^Differences between groups were analyzed by Student’s *t*-test *P = 0.0584*

^d^Body Temperature≧40 °C,^e^ Body Temperature≧41 °C

^f^One death occurred on the third day after challenge. The presence of *S. suis* serotype

1 bacteria was confirmed by bacteriology and multiplex PCR

**Table 2:** Fever and bacteremia of pig after challenge with homologous *Streptococcus suis* serotype 2 bacteria

Group^a^	Cases of fever / bacteremia^b^ (%)							
	Day 0	1	2	3	4	5	6	7
Vaccination^c^	0/0	33/0	0/0	0/0	33/0	0/0	0/0	0/0
Control	0/0	33^d^/0	33 /0	0/0	0/0	0/0	0/0	0/33

^a^Vaccination: *S. suis* 2 + rSao, Control: sterile saline

^b^The fever and bacteremia of piglets were monitored daily for 7 days after challenge

^c^Differences between groups were analyzed by Student’s *t*-test *P =* 0.140

^d^Body Temperature≧40 °C

### Serum antigen-specific antibody titers

**Fig. 2:**
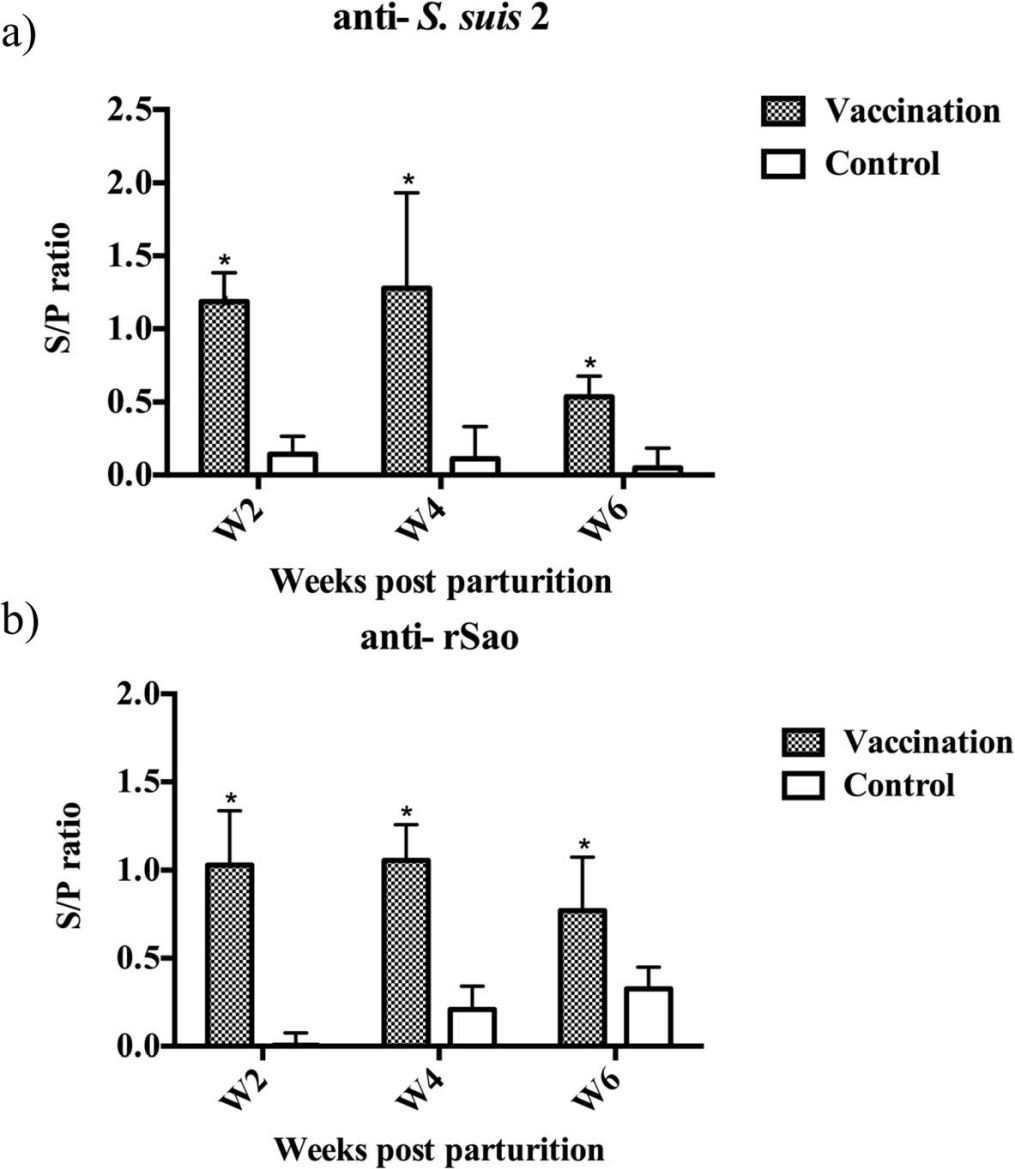
The antibody response of (**a**) *S. suis* 2 (**b**) rSao-specific IgG in the serum of piglets from sows with or without (*n* = 5 each) prepartum immunization with inactivated *S. suis* 2 + rSao. Data are represented as Mean ± SEM of peak absorbance values as determined by indirect ELISA performed on diluted (1:500) serum samples. S/P ratio = (sample – Nˉx) / (PCˉx – NCˉx). Differences between groups were analyzed by Student’s *t*-test, statistically significant difference (*P <* 0.05) in comparison to the control group

Results indicated that antigen-specific antibodies, IgG against *S. suis* serotype 2 bacteria and rSao, in serum were significantly (*P* < 0.05) increased in piglets born from vaccinated sows in comparison with those in piglets born from the unvaccinated sows at 2, 4, and 6 weeks after parturition (Fig. 2a and b). The titer of serum IgG against *S. suis* 2, but not rSao, was remarkably reduced for piglets 6 weeks of age.

### Serum cytokine levels

**Fig. 3:**
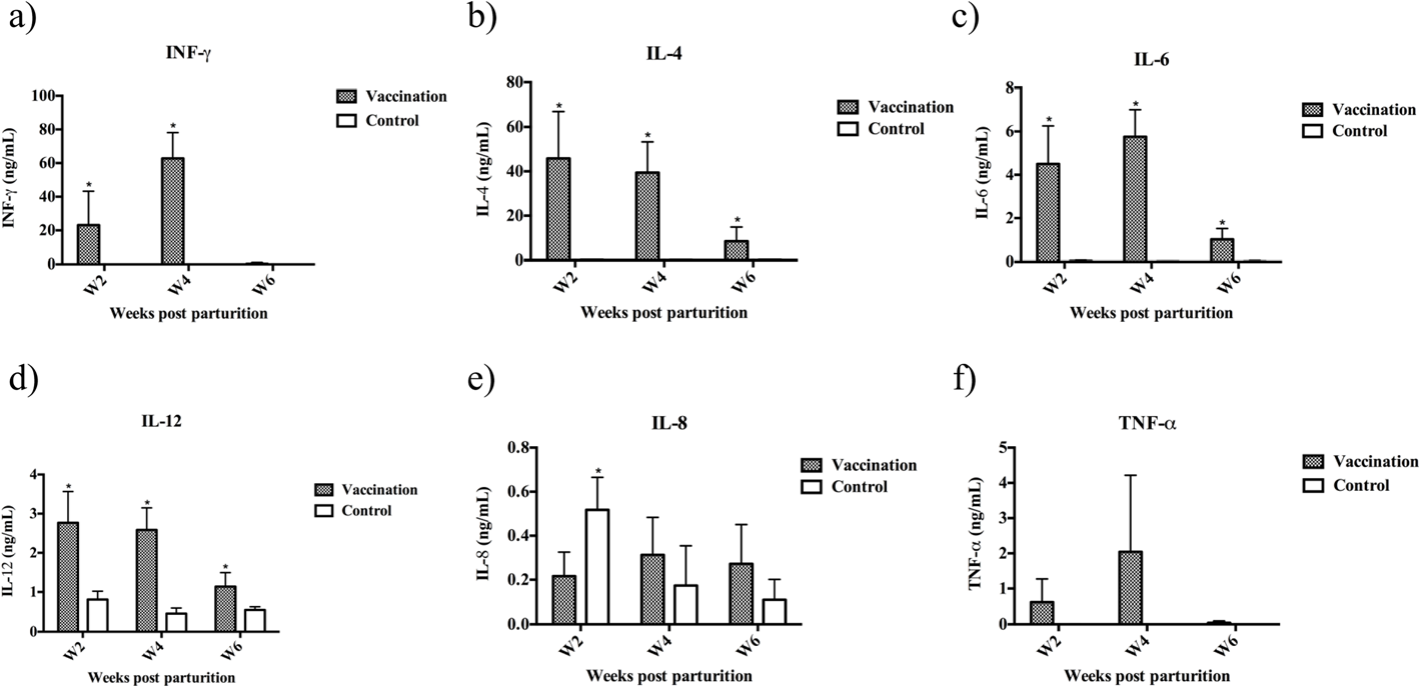
The concentration of cytokine (**a**) IFN-γ (**b**) IL-4 (**c**) IL-6 (**d**) IL-12 (**e**) IL-8 (**f**) TNF-α in piglets from sows with or without (*n* = 5 each) prepartum immunization with inactivated *S. suis* 2 + rSao. Data are represented as Mean ± SEM of the serum concentration of each cytokines determined by MILLPLEX® MAP Kit. Differences between groups were analyzed by Student’s *t-*test, statistically significant difference (*P <* 0.05) in comparison to the control group

The concentration of IFN-*γ* in serum was significantly (*P < 0.05*) increased in the piglets from vaccinated sows at 2 and 4 weeks of age when compared to that of piglets from unvaccinated sows (Fig. 3a). Whereas the concentrations of IL-4 (Fig. 3b), IL-6 (Fig. 3c), and IL-12 (Fig. 3d) were significantly (*P < 0.05*) elevated in piglets from vaccinated sows at 2, 4, and 6 weeks after parturition when compared to those of piglets from unvaccinated sows. In contrast, piglets from vaccinated sows had a significantly (*P < 0.05*) higher concentration of IL-8 at 2, but not 4 and 6 weeks of age (Fig. 3e), when compared to piglets from vaccinated sows. In the case of TNF-α, no differences were observed between the two groups at all the time points (Fig. 3f).

### Clinical symptoms and pathological lesions after sacrifice

According to the pathological examination, piglets from sows in the control group showed various clinical signs, including fever, anorexia, and gross lesions in the peritoneal cavity, joints and lungs, in response to the challenge. In contrast, piglets from sows in the vaccinated group remained healthy and only mild lesions were observed. Results from the lesion score indicated that prepartum vaccination in sows significantly (*P* < 0.05) reduced the lesion score by 75% (7 v.s. 27) and 81% (4 v.s. 21) in their piglets after challenge with *S. suis* serotype 1 and 2 bacteria, respectively (Tables 3 and 4). Presence of *S. suis* bacteria in the necropsy samples of various organs (synovial fluid, peritoneal cavity and lungs) was verified and confirmed, using multiplex PCR, to be the *S. suis* serotype 1 and 2 strains used for challenge (Tables 1 and 2).

**Table 3:** Percentage of pathological lesion score after challenge with heterologous *Streptococcus suis* serotype 1 bacteria

Group^a^	Total Score	Reduced Percentage	The Score of Gross Lesions^b^					
			Brain	Serosa	Joint	Spleen	Liver	Lung
Vaccination	7	75%^c^	1 (1/3)	0 (0/3)	3 (1/3)	0 (0/3)	0 (0/3)	3 (1/3)
Control^d^	27	**-**	1 (1/3)	7 (3/3)	7 (3/3)	1 (1/3)	4 (2/3)	7 (3/3)

^a^Vaccination: *S. suis*2 + rSao, Control: sterile saline

^b^No lesion = 0, lesion area <33% = 1, lesion area 33–66% = 3, lesion area >66% = 5

^c^Differences between groups were analyzed by Student’s *t*-test *P* < 0.05

^d^The number of piglets in a group that was confirmed to have the presence of *S*

*suis* serotype 1 bacteria

**Table 4:** Percentage of pathological lesion score after challenge with homologous *Streptococcus suis* serotype 2 bacteria

Group^a^	Total Score	Reduced Percentage	The Score of Gross Lesions^b^					
			Brain	Serosa	Joint	Spleen	Liver	Lung
Vaccination	4	81.0%^e^	0 (0/3)	0 (0/3)	0 (0/3)	0 (0/3)	0 (0/3)	4 (2/3)
Control^d^	21	**-**	0 (0/3)	0 (0/3)	0 (0/3)	5 (3/3)	3 (3/3)	13 (3/3)

^a^Vaccination: *S. suis*2 + rSao, Control: sterile saline

^b^No lesion = 0, lesion area <33% = 1, lesion area 33– 66% = 3, lesion area >66% = 5

^c^Differences between groups were analyzed by Student’s *t*-test *P* < 0.05

^d^The number of piglets in a group that was confirmed to have the presence of *S*

*suis* serotype 2 bacteria

### Histopathological examination

**Fig. 4:**
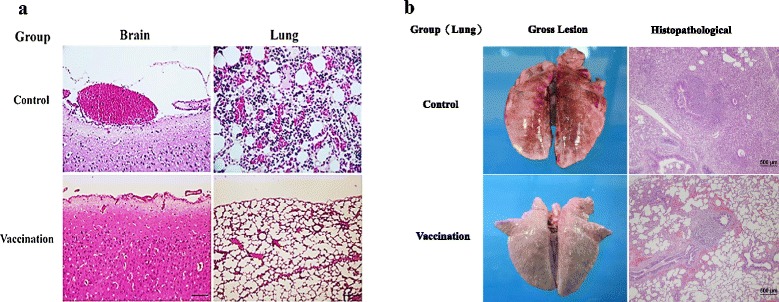
Results of histopathological examination. Piglets from sows with or without prepartum immunization with inactivated *S. suis* 2 + rSao were challenged with (**a**) heterologous *S. sui* serotype 1 and (**b**) homologous *S. sui* serotype 2 bacteria at the 5 weeks of age. The histopathological examination was performed on the 7^th^ day after the challenge. Control pigs manifested severe brain and lung lesions, including slight vascular congestion on the brain meninges and shedded epithelial cells in the lung alveolar space

All piglets survived from challenge were sacrificed and dissected on the 7^th^ day after challenge. Thereafter, histopathological examination was performed. Piglets from unvaccinated sows displayed signs of lesions in the brain and lung, including slight vascular congestion on the brain meninges, influx of neutrophils, lymphocytes, and shedded epithelial cells in the lung alveolar space, and accumulation of neutrophils in the bronchium cavity, indicating the development of bronchopneumonia, hemorrhagic bronchial pneumonia, and lung abscess (Fig. 4a and b). In contrast, above-mentioned signs of lesions were not observed in piglets from vaccinated sows after dissection.

## Discussion

Piglets are more susceptible to infections due to the stress associated with weaning and the immature immune system during the early stage of life. Prepartum immunization in sows has been proven safe and efficiently conveys protection to piglets through the transfer of passive immunity in milk. Prepartum vaccination with a subunit vaccine containing recombinant *Pasteurella multocida* toxin proteins as the antigen in sows was able to enhance the level of neutralizing antibodies, reduce the degree of turbinate atrophy, and increase the survival rate in piglets with experimentally induced progressive atrophic rhinitis (PAR) [20]. Immunizing pregnant sows (52 and 32 days before parturition, respectively) with a type O1 foot and mouth disease oil emulsion vaccine significantly increased the serum level of neutralizing antibodies in piglets 3 days after birth [21]. Moreover, 3-week-old piglets born from sows immunized with a classical swine fever vaccine (E2) twice after insemination had significantly higher maternal antigen-specific antibodies as indicated by the ND_50_ (neutralization dilution 50%) [22].

We previously demonstrated that immunization with the rSao (from serotype 2 *S. suis*) protein expressed by bioreactors formulated with w/o/w adjuvant increased antigen-specific antibody titers, the percentages of CD8^+^ and CD4^+^/CD8^+^ double-positive T cells, and successfully provided cross-serotype protection against the challenge of heterologous *S. suis* serotype 1 bacteria in pigs [19]. In combination with Quil A, rSao can reduce clinical symptoms after challenge infection with heterologous *S. suis* and stimulates strong opsonizing antibody responses [18]. A previous study has indicated that the pregnant sows immunized with a PAR vaccine containing three short fragments of recombinant subunit *Pasteurella multocida* toxin (rsPMT) in combination with *Pasteurella multocida* type A bacterin (rsPMT + *P. multocida* bacterin) as the antigens can elicit higher neutralizing antibody titer in colostrum than pregnant sows immunized with the vaccine containing rsPMT only (1:101 v.s. 1:80) [20]. The immunization protected 60% of piglets born from these sows after challenged by intramuscular injection with five-fold lethal dose of authentic PMT. The results implied that combination of recombinant antigen with inactivated bacteria as the antigen may enhance the immune responses elicited by the vaccine. In addition, virulence factors of *S. suis* other than Sao, such as suilysin, muramidase-released protein (MRP), and extracellular factor (EF), also play a role in the pathogenesis of *S. suis*. Immunization with a subunit vaccine containing these virulence factors (MRP^+^/EF^+^) from *S. suis* serotype 2 as the antigens has been demonstrated to protect pigs from challenge of homologous or heterologous *S. suis* strains [25]. Addition of inactivated *S. suis* bacteria containing these surface virulent factors into the vaccine formula may provide extra antigens and elicit broader protection in immunized animals. Therefore, pregnant sows were immunized with the vaccine containing inactivated *S. suis* serotype 2 plus rSao as the antigens and the passive immunity against heterologous *S. suis* serotype 1 infection in their piglets was investigated in the present study.

The serum titer of IgG against rSao and *S. suis* serotype 2 in piglets from vaccinated sows remained significantly higher than that of piglets from unvaccinated sow until 6 weeks of age (Fig. 2a and b). After challenge with live *S. suis* serotype 1 bacteria, the lesion score of piglets from vaccinated sows was reduced by 75%. These results implied that immunization with rSao plus inactivated *S. suis* serotype 2 in pregnant sows enhanced the passive immunity against *S. suis* and provided cross-protection in their piglets. The Sao protein is highly conserved among *S. suis* strains and Sao-specific antibodies have been shown to cross-react with 28 different serotypes of *S. suis* [13]. The lesion score was reduced by 60% in pigs immunization with rSao alone after challenge with live heterologous *S. suis* bacteria [19]. Results from the present study revealed that the lesion score was reduced by 75% in piglets born from sows immunized with inactivated *S. suis* bacteria plus rSao after challenge with live heterologous *S. suis* bacteria.

In piglets produced by vaccinated sows, the antigen-specific IgG titer remained high until 6 weeks of age (Fig. 2a and b). This is in agreement with a previous study in which pregnant sows were immunized with the autogenous *S. suis* bacterin and MRP-specific IgG remained detectable until 6 weeks of age [25]. However, a remarkable reduction of the antigen-specific IgG titer in serum was observed in the 6 weeks of age in comparison with the titers in the 2 and 4 weeks of age, which is possibly due to the half-life of IgG absorbed from colostrum. The IgG concentrations in piglets at 24 h of age ranged from 18.7 to 39 mg/mL and gradually decreased to 6.3 mg/mL at 5 to 6 weeks of age [26]. The w/o/w adjuvant used in our vaccine has been shown to stimulate both humoral and cell-mediated arms of immune responses [17, 23].

Compared to piglets from sows in the control group, piglets from sows in the vaccinated group had significantly (*P* < 0.05) increased levels of IL-4, IL-6, IL-12, and IFN-γ in serum after parturition. The source of these cytokines in piglets’ circulation could be mainly from the absorption of cytokines in colostrum before gut closure. IL-4 and IL-6 plays a role in enhancing Th2-type immune responses, B cell proliferation, and antibody production [27]. On the other hand, IL-12 and IFN-γ are the key cytokines that lead immune responses to Th1-type. IL-12 activates immune cells, such as NK cells and naïve CD4^+^ T cells, to secret IFN-γ, which in turn facilitate macrophages maturation [28–32]. Therefore, the higher concentration of cytokines in serum may contribute, at least partially, to the enhanced resistance to challenge with live *S. suis* bacteria in piglets from vaccinated sows. It is unclear why the level of serum IL-8 was increased only at 2 weeks after parturition in piglets from sows in the control group. IL-8 is a potential chemoattractant that is up regulated during the course of intramammary infections [33]. However, whether the sows had mastitis was not determined in our study. The serum levels of TNF-α were low and not different between the two groups, which is probably due to the short half-life of this cytokine [27].

Results of the histopathological examination revealed that piglets from unvaccinated sows had slight vascular congestion on the brain meninges, influx of neutrophils, lymphocytes, and shedded epithelial cells in the lung alveolar space, and signs of suppurative bronchopneumonia, hemorrhagic bronchial pneumonia, and lung abscess (Fig. 4a and b). In piglets i.v. challenged by live *S. suis*, the main lesions (found in the joints, spleen, liver, and kidneys) are associated with synovitis, spleen inflammation, hepatitis, and pneumonia [25]. Dissection of the piglet that died on the third day after challenge showed severe lesions in the lung. In contrast, lesions were not found in any of the piglets from sows in the vaccinated group after dissection, indicating that the enhanced passive immunity against *S. suis* was sufficient to protect piglets from *S. suis* challenge.

## Conclusions

Prepartum immunization of inactivated *S. suis* bacterin plus rSao in sows was able to increase serum antigen-specific antibody titers, the concentrations of IFN-γ, IL-4, IL-6 and IL-12 in serum, and provide cross-protection against *S. suis* infections in the piglets. The protection was characterized by the reduction on lesion score (75 and 81%), bacteremia, and fever. This vaccine and immunization approach may be applied to enhance the protection against both heterologous and homologous *S. suis* infections in newborn piglets.

## Abbreviations

Abpb: Amylase-binding protein B
ANOVA: Analysis of variance
BHI: Brain-heart infusion
BSA: Bovine serum albumin
CFU: Colony-forming unit
CPS: Capsular polysaccharides
DNA: Deoxyribonucleic acid
*E. coli*: Escherichia coli
EF: Extracellular factor
ELISA: Enzyme-linked immunosorbent assay
i.m: Intramuscular
i.v.: Intravenous
IFN-γ: Interferon-gamma
IgG: Immunoglobulin G
IL: Interleukin
IPTG: Isopropyl-β-d-thiogalactoside
LPXTG: Leu-Pro-X-Thr-Gly
MRP: Muramidase-released protein
ND_50_: Neutralization dilution 50%
NK cell: Natural killer cell
PAR: Progressive atrophic rhinitis
PCR: Polymerase chain reaction
rsPMT: Recombinant *Pasteurella multocida* toxin
*S. suis*: *Streptococcus suis*
S/P: Sample to positive ratio
Sao: Surface antigen one
SAS: Statistical analysis system
SDS-PAGE: Sodium dodecyl sulfate-polyacrylamide gel electrophoresis
SsnA: Surface-anchored DNA-nuclease
Th: T-helper cell
TNF-α: Tumor necrosis factor-alpha
w/o/w: Water in oil in water
